# “I Call It Health Science, Not Nursing”: Male International Students Balancing Nursing Career Aspirations With Cultural Expectations

**DOI:** 10.1177/10436596251337062

**Published:** 2025-04-30

**Authors:** Animesh Ghimire, Yunjing Qiu

**Affiliations:** 1 Monash University, Clayton, Victoria, Australia; 2 Sustainable Prosperity Initiative Nepal, Kathmandu, Nepal; 3 University of Technology Sydney, Ultimo, New South Wales, Australia

**Keywords:** cultural stigma, gender stereotypes, international nursing students, hostility, nursing education, South Asian

## Abstract

**Introduction:**

Male South Asian international nursing students in Australia navigate a complex interplay of cultural expectations, personal aspirations, and gender stereotypes. These students often encounter stigma and familial pressure due to their pursuit of a non-traditional career in nursing.

**Method:**

A qualitative descriptive design involving 11 participants was employed. Data were analyzed using a thematic analysis framework informed by principles of transnationalism, intersectionality, and identity work.

**Results:**

Participants strategically employed linguistic camouflage, referring to their studies as “health science” rather than “nursing.” Motivations for pursuing nursing included migration opportunities, economic considerations, and aspirations for personal freedom and self-acceptance. The term “nurse” carried significant cultural baggage.

**Discussion:**

This study reveals the strategic and pragmatic nature of these students’ career choices. Despite facing unique challenges, including navigating a hostile educational environment, participants demonstrated remarkable resilience. Findings underscore the need for culturally sensitive support programs, curriculum reforms that challenge gender stereotypes, and faculty training in cultural competence to promote inclusivity and empower these students.

## Introduction

Culture, as a complex interplay of shared beliefs, values, and practices, significantly shapes the nursing profession. It influences the perceptions and experiences of those who enter the field and affects their career aspirations, educational journeys, and professional practice. This cultural tapestry is woven with threads of tradition, gender roles, and societal expectations, which can either empower or constrain individuals depending on their alignment with established norms (Rodríguez-Pérez et al., 2022). In nursing, where compassionate care intersects with scientific knowledge, culture acts as a potent force, shaping the very fabric of care (Burns et al., 2020; Cassidy, 2024). This dynamic is particularly evident in regions like South Asia, where cultural norms and societal expectations surrounding gender roles can significantly impact the professional landscape, especially within traditionally female-dominated fields like nursing (Baby, 2023).

South Asia encompasses countries such as Afghanistan, Bangladesh, Bhutan, India, Maldives, Nepal, Pakistan, and Sri Lanka (Joshi, 2015), where nursing is viewed through the lens of gender roles and societal expectations (Baby, 2023; Darr et al., 2008). In this context, the cultural impact is particularly profound for male individuals who challenge the status quo by aspiring to a nursing career (Mohulatsi et al., 2024). Most South Asian societies are characterized by predominantly patriarchal structures, which are hierarchical social systems that often reinforce traditional gender roles (Bussolo et al., 2023). Within these structures, men are typically expected to be the primary breadwinners and occupy positions of authority, while women are often relegated to domestic roles (Sengar & Shah, 2024). This patriarchal context significantly shapes the perception of nursing, which is often regarded as a feminine profession closely tied to caregiving and subservience roles. These roles are often deemed incompatible with traditional notions of masculinity. In this context, masculinity refers to the socially constructed set of attributes, behaviors, and roles generally associated with men within a particular culture. Many South Asian societies often emphasize strength, dominance, stoicism, and breadwinning, contrasting with the perceived feminine qualities of nurturing and emotional expressiveness often associated with nursing (Qureshi et al., 2020). This cultural perception can create a formidable barrier for male students, forcing them to navigate a complex landscape of conflicting expectations and limited opportunities.

Despite these challenges in the South Asian context, Australia has witnessed a notable increase in the representation of men in the nursing profession, currently accounting for 12.1% of the nursing workforce (Australian Nursing and Midwifery Federation, 2024). This rise can be attributed, in part, to the greater social recognition of nursing in Australia, where nurses are consistently ranked as the most trusted profession (Morgan, 2021), combined with the attractive migration opportunities it offers (Migration Affairs, 2024). In addition, the gender pay gap in nursing, which often favors male nurses, serves as an additional pull factor for male international students (International Council of Nurses [ICN], 2022; Woo et al., 2022). Interestingly, a significant proportion of international students in Australia originate from South Asia, with Indians (17%) and Nepalis (8%) being among the most prominent groups (Department of Education, 2024). While these statistics do not specifically identify male students pursuing nursing, they reflect a broader trend of South Asian individuals seeking educational and professional opportunities in Australia. Considering nursing’s well-established route for migration and professional accreditation, coupled with the prospect of enhanced income potential, relatively low tuition costs for international students, and increased opportunities for social mobility, it presents a compelling career pathway for many individuals (Dos Santos et al., 2024; Ghimire & MacDonald, 2025). However, despite the influx of South Asian students, there remains a limited exploration of male South Asian nursing students in the existing literature and how they navigate the cultural complexities associated with nursing as a career choice.

Adapting to a new cultural context, particularly in Western societies with different gender norms and expectations surrounding nursing, could potentially lead to experiences of cultural dissonance for male South Asian international students. These students may find themselves grappling not only with the practical demands of navigating a new educational system and professional environment but also with the psychological and social complexities of reconciling their masculine identities with the traditionally perceived feminine nature of nursing. To manage these challenges, some students employ linguistic framing strategies, such as linguistic camouflage or maneuvering. Linguistic framing refers to how language shapes perceptions and influences how a particular topic or issue is understood. It involves carefully selecting words and phrases to create a specific narrative or frame of reference (Formanowicz & Hansen, 2022). In this context, camouflage refers to concealing one’s true intentions or identity to avoid negative social consequences (Cook et al., 2021). Maneuvering involves strategically navigating social situations, often employing indirect communication or actions, to achieve desired outcomes while minimizing potential conflict or disapproval (Zsuzsanna, 2018). While there is a growing body of research exploring the experiences of male nursing students in Western contexts (DeVito, 2016; Grimshaw et al., 2024; Petges & Sabio, 2020), the unique challenges encountered and the strategies employed by male South Asian international students remain largely unexplored. Specifically, there is a gap in understanding how these students’ experiences intersect with issues of sexuality, societal expectations, family prestige, and this includes the pressure to conform to heteronormative ideals. This gap in the literature obscures the complex interplay of cultural, social, and personal factors that shape their educational and professional journeys, particularly within the domain of transcultural nursing and the negotiation of masculine identities in a predominantly female profession. Furthermore, the existing literature has not adequately addressed the specific push and pull factors that motivate male South Asian students to pursue nursing in Australia, including the role of financial considerations, migration aspirations, and the desire for personal and professional growth. Thus, this study addresses this gap by asking: How do male South Asian international students studying nursing in Australia navigate the cultural complexities associated with their chosen profession, and what strategies do they employ to reconcile personal aspirations with societal expectations?

## Method

### Theoretical Lens

This study is grounded in the theoretical frameworks of intersectionality and identity work, providing a lens through which to understand the complex experiences of male South Asian international nursing students in Australia. Intersectionality, as proposed by Crenshaw (1989), recognizes that individuals hold multiple, interconnected social identities (e.g., gender, race, ethnicity, class, sexuality) that simultaneously shape their experiences and are shaped by systems of power and oppression. In the context of this study, intersectionality helps us understand how the participants’ identities as men, South Asians, international students, and aspiring nurses intersect to create unique challenges and opportunities. Identity work, as described by Caza et al. (2018), refers to the ongoing process by which individuals negotiate and construct their sense of self in relation to their social environment. This framework is particularly relevant for understanding how these students navigate the cultural complexities of their chosen profession, balancing their personal aspirations with societal expectations, both from their home cultures and the host culture. The concept of transnationalism is also relevant, as it acknowledges the experiences of individuals who maintain connections and identities across national borders (Belford & Lahiri-Roy, 2019). It provides a framework for understanding how these students negotiate their sense of belonging in their home and host countries. These theoretical perspectives informed the research questions, data collection, and analysis, guiding the exploration of how participants’ intersecting identities shape their experiences and strategies for navigating a traditionally female-dominated field in a new cultural context.

### Ethical Consideration

This project received ethics approval from the Monash University Human Research Ethics Committee (MUHREC), project ID 45088, following a full review of the research protocol. Recognizing that international students are considered vulnerable due to potential power imbalances and cultural factors (Deuchar, 2022), we took several measures to ensure their voluntary and informed participation. Initial verbal consent was secured to assess participant interest, followed by distributing a detailed Plain Language Statement Form (PLSF) outlining the study’s purpose, procedures, potential risks and benefits, and their right to withdraw at any time without penalty. Participants were given ample time to review the PLSF and ask any questions before providing written consent, which was obtained prior to the interview. To maintain anonymity, pseudonyms were used throughout the study, and any potentially identifying information was removed from the transcripts. Confidentiality was ensured by storing all data securely and limiting access to the research team only.

### Study Design

This study employed a qualitative descriptive design, chosen for its suitability in exploring the lived experiences and perceptions of male South Asian international nursing students in Australia. This methodological approach enabled the researchers to capture the richness and complexities of the participants’ narratives, preserving the authenticity and significance of their voices (Doyle et al., 2020). Qualitative description aligns well with the theoretical frameworks of intersectionality and identity work as it allows for an in-depth examination of how individuals’ social identities and their negotiations of these identities shape their experiences (Lambert & Lambert, 2012). Qualitative description distinguishes itself from other qualitative methodologies such as grounded theory, which seeks to formulate new theoretical frameworks, and phenomenology, which delves into the fundamental nature of lived experiences. Instead, qualitative description prioritizes a thorough and straightforward characterization of events, articulating them in accessible language to capture the essence of those occurrences effectively. This approach is particularly useful, as in this study, the goal is to understand a phenomenon that has not been extensively researched, where existing knowledge is limited and requires a detailed descriptive foundation. An inductive approach was used to explore how male South Asian international nursing students navigate cultural complexities and challenges within the nursing profession. The Consolidated Criteria for Reporting Qualitative Research (COREQ) guidelines were adhered to throughout the research process to ensure rigor and transparency (Tong et al., 2007).

### Sample

The study’s sample consists of male South Asian international students currently enrolled in their final semester of a bachelor in nursing program at an Australian university. Participants were recruited through a combination of purposive and snowball sampling techniques (Palinkas et al., 2015). Purposive sampling identified potential participants who met the inclusion criteria, while snowball sampling leveraged existing networks within the South Asian student community to identify additional participants. Recruitment presented challenges due to the sensitive nature of the research topic and the limited pool of potential participants who met all inclusion criteria. To overcome this, we utilized the first author’s (AG) established connections within the South Asian student community to foster trust and rapport with prospective participants, underscoring our commitment to confidentiality and anonymity. Preliminary communications were conducted via email, subsequently complemented by in-person meetings to provide a more comprehensive overview of the study’s objectives and methodologies.

Inclusion criteria consisted of (a) identifying as male gender, (b) South Asian origin (from Afghanistan, Bangladesh, Bhutan, India, Maldives, Nepal, Pakistan, and Sri Lanka), (c) currently enrolled in the final semester of a 3-year bachelor in nursing program at an Australian university, and (d) willing and able to participate in an in-depth interview. Focusing on students in their final semester allowed us to capture their experiences and perspectives at a crucial juncture in their academic and professional journeys. These students are nearing the completion of their nursing education and are contemplating their transition into the workforce. Their insights into the challenges and strategies they have employed to navigate cultural complexities and gendered expectations throughout their studies will be particularly valuable for understanding the interplay between culture, gender, and career aspirations.

### Data Collection

Data were collected by the first author (AG) through semi-structured interviews designed to elicit detailed and nuanced narratives about participants’ thoughts, experiences, attitudes, and beliefs (DeJonckheere & Vaughn, 2019). The researchers had no prior relationship with the participants. After obtaining informed consent, each participant engaged in an interview that lasted approximately 40–60 minutes. The interview was conducted in the public library at a time suitable for the participants. The interview protocol explored the participants’ experiences navigating cultural complexities and challenges as male South Asian international nursing students in Australia. Key areas of exploration included their perceptions of nursing within their home cultures, their experiences of cultural dissonance and adaptation, strategies they employ to manage expectations from family and peers, and their overall sense of belonging within the nursing profession (Table 1).

**Table 1.** Interview Questions.

No.	Interview questions
1	Can you describe your initial motivations for choosing nursing as a career path?
2	How are nurses perceived in your home country?
3	Have you experienced any challenges or conflicts related to your decision to study nursing, particularly from family or friends back home?
4	How do you typically describe your field of study to people in your home country?
5	Can you share any specific instances where you felt the need to modify your language or avoid certain terms when discussing your studies with people from your home country?
6	How has your experience studying nursing in Australia influenced your perceptions of the profession and its role in society?
7	What are your career aspirations after graduation?
8	Do you feel a sense of belonging within the nursing profession in Australia?
9	How do you perceive the role of gender in shaping your experiences as a nursing student in Australia?
10	What advice would you give to other male South Asian students considering a career in nursing?

An integrated methodology for data collection was employed to maintain data integrity and fortify the validity of the resultant interpretations. The researchers documented their observations and reflections through memos and field notes. This triangulation of data sources facilitated a more comprehensive understanding of the participants’ perspectives, feelings, and experiences, thereby enhancing the trustworthiness and validity of the study’s findings.

### Data Analysis

Data analysis was conducted using Braun and Clarke (2023) thematic analysis framework. The analysis involved thoroughly examining the interview transcripts from the eleven participants. Several measures were implemented to enhance the trustworthiness of the data. Each interview was audio-recorded and transcribed verbatim by the first author. All participants were then invited to review a summary of their interview and the corresponding transcript to ensure accuracy and address any potential misunderstandings. This member-checking process further strengthened the credibility and authenticity of the findings.

The analysis aims to achieve both code and thematic saturation (Naeem et al., 2024; Rahimi & Khatooni, 2024). Code saturation was reached when no new codes emerged from the data, indicating that the coding framework comprehensively captured the range of concepts and ideas expressed by the participants. Thematic saturation was achieved when no new themes or sub-themes were identified, and further analysis did not yield any new insights or perspectives, suggesting that the themes adequately represented the overarching patterns and complexities within the data. While related, these processes are distinct. Code saturation focuses on the breadth of concepts captured, while thematic saturation focuses on the depth and comprehensiveness of the overarching narrative. Code saturation was determined after analyzing nine interviews, where no new codes emerged. Thematic saturation was confirmed after analyzing the eleventh interview, where no new themes or sub-themes were identified, and the existing themes were sufficiently rich and nuanced to explain the data. Table 2 provides a detailed overview of the data analysis process, illustrating the iterative movement from meaning units to codes, categories, sub-themes, and overarching themes. The table demonstrates how specific excerpts from the interviews (meaning units) were assigned codes that captured their essential meaning. These codes were then grouped into broader categories based on shared characteristics. Sub-themes emerged from the analysis of the categories, capturing the nuances of the participants’ experiences. Finally, the overarching themes were developed to represent the study’s key findings. For example, the initial code “linguistic camouflage” was later subsumed under the broader theme of “The ‘Health Science’ Facade: Navigating Cultural Stigma as Male Nursing Students,” which captured the strategic use of language to manage cultural expectations. Another example is the code “migration opportunity,” which contributed to the theme “Calculated Choices, Complex Journeys: Migration, Masculinity, and the Pursuit of Nursing Careers,” reflecting the pragmatic motivations behind the participants’ career choices. Table 2 provides a detailed overview of the data analysis process.

**Table 2.** Data Analysis Process.

Meaning unit	Code	Category	Sub-theme	Theme
“In my country, nursing is seen strictly as a woman’s job.”—Arun	Gendered profession	Cultural perceptions	Stigma and shame	The “Health Science” Façade: Navigating Cultural Stigma as Male Nursing Students
“Admitting that I’m studying nursing would invite ridicule and disappointment from friends and family.”—Arun	Societal disapproval	Cultural expectations	Fear of judgment	The “Health Science” Façade: Navigating Cultural Stigma as Male Nursing Students
“By calling it ‘health science,’ I preserve their respect and can pursue my ambitions without conflict.”—Arun	Linguistic camouflage	Coping strategies	Strategic reframing	The “Health Science” Façade: Navigating Cultural Stigma as Male Nursing Students
“Nursing is my golden ticket to Australia.”—Vikram	Migration opportunity	Motivation	Economic advancement	Calculated Choices, Complex Journeys: Migration, Masculinity, and the Pursuit of Nursing Careers
“It’s a chance to build a stable future for myself and my family.”—Vikram	Future security	Motivation	Family obligations	Calculated Choices, Complex Journeys: Migration, Masculinity, and the Pursuit of Nursing Careers
“It’s not just about the job; it’s about freedom, about living without fear.”—Kamal	Personal liberation	Motivation	Self-actualization	Calculated Choices, Complex Journeys: Migration, Masculinity, and the Pursuit of Nursing Careers
“Nursing is a means to an end. Once I have my permanent residency, I’ll go back to school, maybe study business or law—something that commands respect back home.”—Ruvin	Stepping stone	Career aspirations	Status and recognition	Calculated Choices, Complex Journeys: Migration, Masculinity, and the Pursuit of Nursing Careers
“It is a common practice that other friends who are studying nursing do the same. They do not utter the word ‘nurse’ in front of extended friends and family.”—Amir	Shared strategy	Coping mechanisms	Collective identity	“Nurse,” a Word Unspoken: Linguistic Camouflage and the Quest for Cultural Acceptance
“I’m not just studying ‘nursing’; I’m becoming a healer, a caregiver, a vital part of the healthcare team. It’s time we redefine what it means to be a man in the 21st century.”—Ishaan	Redefining masculinity	Challenging norms	Personal agency	“Nurse,” a Word Unspoken: Linguistic Camouflage and the Quest for Cultural Acceptance
“Back home, men are expected to become doctors, engineers, or businessmen—professions deemed suitable for males.”—Farhan	Traditional expectations	Cultural perceptions	Societal pressure	The “Health Science” Façade: Navigating Cultural Stigma as Male Nursing Students
“The term ‘nurse’ carries a significant stigma where I’m from; only females study nursing.”—Rajesh	Nursing stigma	Cultural perceptions	Gender stereotypes	The “Health Science” Façade: Navigating Cultural Stigma as Male Nursing Students
“Once I have my permanent residency, I can pursue further studies in healthcare management or public health.”—Ruvin	Future aspirations	Career aspirations	Professional development	Calculated Choices, Complex Journeys: Migration, Masculinity, and the Pursuit of Nursing Careers
“I’m proud of my choice to become a nurse, but I’m also aware of the cultural barriers I face because of my choice.”—Amir	Pride and challenges	Personal reflection	Navigating cultural barriers	“Nurse,” a Word Unspoken: Linguistic Camouflage and the Quest for Cultural Acceptance
“Just by labeling my field as ‘health science,’ it helps maintain my family’s pride while I navigate my own career path.”—Rajesh	Maintaining family pride	Coping strategies	Protecting image	The “Health Science” Façade: Navigating Cultural Stigma as Male Nursing Students

### Rigor and Reflexivity

This qualitative study acknowledges the inherent subjectivity in interpretive research and emphasizes the importance of researcher reflexivity in ensuring the trustworthiness of the findings (Peddle, 2022). The research team, comprising experienced qualitative researchers with diverse backgrounds and expertise in cultural studies and nursing education, actively engaged in critical self-reflection throughout the research process. This reflexivity involved acknowledging and examining their own cultural backgrounds, assumptions, and potential biases, which could shape both the research process and the interpretation of data. The first author (AG) is from a South Asian background, is a former international student, and is a migrant nurse who identifies as male. The second author (YQ) is from an East Asian background, is a former international student, and is a migrant nurse.

The first author, who conducted the interviews and initial data analysis, has a background in cultural studies and extensive experience conducting qualitative research with diverse populations. Their expertise in cultural sensitivity and qualitative methodologies ensured a rigorous data collection and analysis approach. Their identity as a South Asian male and former international students provided a unique insider perspective, allowing them to establish rapport with participants and understand the nuances of their experiences. However, they were also aware of the potential for shared experiences to lead to assumptions or biases. To mitigate this, they maintained a reflexive journal throughout the study, documenting their thoughts, feelings, and reactions to the data. This allowed them to critically examine how their background might influence their interpretations. However, to mitigate potential biases, they engaged in ongoing dialogue with the second researcher, a former international student, and a migrant nurse. The second author, a female with their East Asian background, brought a different cultural lens to the analysis, challenging the first author’s interpretations and ensuring that the findings were not solely reflective of a South Asian perspective. This cross-cultural dialogue enriched the analysis and enhanced the credibility of the findings. This collaborative approach fostered critical discussion and reflexivity, minimizing the influence of individual perspectives and enhancing the study’s rigor.

Furthermore, the research team employed independent coding of the interview transcripts and engaged in collaborative discussions to reach a consensus on emerging themes and interpretations. This strategy ensured that the findings were grounded in the data and not unduly influenced by individual perspectives. While acknowledging the limitations inherent in any qualitative study, the researchers believe that their commitment to reflexivity, coupled with the diverse perspectives and experiences within the team and adherence to established qualitative research standards, contributes to the credibility and meaningfulness of the findings within the context of male South Asian international nursing students in Australia.

## Results

### Participants Characteristics

Eleven male South Asian international students participated in this study. The participants were aged between 23 and 27 years. All were enrolled in the final semester of their bachelor in nursing program. While all participants shared the common experience of being South Asian international students pursuing a nursing career in Australia, their motivations for choosing nursing varied. For some, nursing was a first-choice career path driven by a genuine desire to care for others and contribute to health care. For others, nursing was a strategic pathway to migration and broader career opportunities in Australia. This diversity in motivation highlights the complex interplay of personal aspirations, cultural influences, and pragmatic considerations that shape these students’ educational and professional journeys. Table 3 provides a snapshot of the participants’ socio-demographic characteristics.

**Table 3.** Participant’s Socio-Demographic Characteristics.

Pseudonym	Country of origin	Age	Nursing as first choice
Vikram	India	24	No
Asif	Pakistan	26	No
Arun	Nepal	25	Yes
Farhan	Maldives	23	No
Rajesh	Nepal	27	No
Kamal	Bhutan	24	Yes
Rohan	Nepal	25	No
Ruvin	Sri Lanka	26	No
Raj	India	23	Yes
Amir	India	27	Yes
Ishaan	Bangladesh	24	No

### Findings

Our analysis revealed a complex tapestry of experiences, strategies, and aspirations among male South Asian international nursing students in Australia. These individuals navigate a unique cultural landscape, strategically managing perceptions through linguistic choices and making calculated decisions about their future. Their journeys are marked by a desire for professional recognition, personal freedom, and a redefinition of masculine identity within a traditionally female-dominated field. Three overarching themes emerged from the data to capture these experiences, each reflecting a critical aspect of their journey.

### The “Health Science” Facade: Navigating Cultural Stigma as Male Nursing Students

In many South Asian societies, traditional gender roles are deeply ingrained, often dictating the career paths deemed appropriate for men and women. Male students from these regions who pursue nursing abroad, such as in Australia, frequently find themselves at the intersection of personal aspiration and cultural expectation. This tension directly reflects their intersecting identities as men, South Asians, and aspiring nurses, highlighting the complexities identified by the framework of intersectionality.

In my country, nursing is seen strictly as a woman’s job. Admitting that I’m studying nursing would invite ridicule and disappointment from friends and family. By calling it “health science,” I preserve their respect and can pursue my ambitions without conflict.—Arun

To reconcile this tension and mitigate potential familial and societal disapproval, they often adopt linguistic strategies, such as referring to their field of study as “health science” when communicating with family and friends back home. This reframing serves to mitigate societal stigma, allowing them to pursue their passions while maintaining familial respect.

Back home, men are expected to become doctors, engineers, or businessmen—professions deemed suitable for males. Nursing is considered too feminine[. . .] Describing my studies as “health science” provides a buffer against judgment and allows me to follow my passion privately.—Farhan

This strategic use of language is a form of identity work, as these students actively negotiate their identities as nursing students with the masculine ideals prevalent in their home cultures. By invoking the broader, more socially acceptable term of “health science,” they create a space to pursue their professional goals without directly challenging deeply entrenched cultural norms.

The term “nurse” carries a significant stigma where I’m from; only females study nursing. Moreover—it’s associated with low status and little respect[. . .] Just by labeling my field as “health science,” it helps maintain my family pride while I navigate my own career path.—Rajesh

This act can also be seen as a transnational negotiation of identity, where individuals adapt and modify their self-presentation across different cultural contexts. They are, in essence, managing their identity across borders, presenting a more culturally acceptable narrative to their families while pursuing a path that aligns with their personal aspirations in their host country.

### Calculated Choices, Complex Journeys: Migration, Masculinity, and the Pursuit of Nursing Careers

The decision of male South Asian international students to pursue nursing in Australia is often strategic. Traditional gender roles in South Asian societies typically relegate nursing to a female-dominated profession, casting it as an unsuitable profession for men. However, the prospects of job security, pathways to permanent residency, and increased personal freedom compel these students to carefully navigate cultural stigmas and familial expectations.

Nursing is my golden ticket to Australia. Here, nurses are in high demand. It is a clear path to permanent residency and a chance to build a stable future for myself and my family.—Vikram

This statement exemplifies the concept of transnationalism, as Vikram highlights how nursing in Australia serves as a pathway to achieving broader migration goals. His choice is not solely based on immediate career aspirations but is intertwined with a long-term plan encompassing securing residency and building a future in a new country.

I tried accounting, but the job market was saturated. With nursing, I knew I’d have job security, a good income, and a chance to work in my field. It isn’t my dream career, but it’s a practical choice that gives me a foothold in this country.—Asif

Asif’s experience further illustrates this transnational decision-making process. He strategically chose nursing to achieve economic stability and establish himself in Australia, not necessarily out of passion for the field. This highlights how these students make calculated choices across national borders, leveraging opportunities in one country to achieve long-term goals. However, these calculated choices are not without their challenges.

I understand the need to learn clinical skills, but repeatedly telling me that my accent is hard to understand made me feel like an outsider. It has nothing to do with my clinical knowledge but something that I cannot fix[. . .] The preceptor gave more attention to the domestic student and was critical of me due to my communication style not matching their idea of “ideal communication.”—Raj

Raj’s experience brings to light the often-overlooked hurdles faced by international students in clinical settings. Despite their strategic career choices, they encounter systemic biases that can hinder their professional development and sense of belonging. This highlights the need to move beyond a purely economic lens when examining transnational migration and consider the lived experiences of individuals navigating new and sometimes challenging environments.

Back home, my sexuality is taboo. I couldn’t live openly, couldn’t be myself. Nursing offered me a way out, a good career, a profession that is accepting of people like me, and an option to live in Australia permanently. It’s not just about the job; it’s about freedom [. . . ], living without fear [. . .].—Kamal

Kamal’s narrative introduces another dimension to these calculated choices, revealing how personal freedom and self-acceptance can be intertwined with migration aspirations. His desire to live openly and authentically, unconstrained by the social norms of his home country, further underscores the transnational nature of his journey.

My family always dreamed of me becoming a doctor. However, medicine is so competitive, and I could not get a scholarship back home. Hence, I came to Australia. Nursing offered a faster route to a stable career and a chance to help people. Once I have my permanent residency, I can pursue further studies in healthcare management or public health. It’s a stepping stone to a career that will make my family proud.—Rohan

Rohan’s statement reinforces the idea of nursing as a “stepping stone” within a larger transnational plan. He perceives nursing as a strategic path toward obtaining permanent residency, which will facilitate his pursuit of alternative education and enable him to cultivate a career that meets the aspirations and expectations set forth by his family. Similarly, another participant highlighted:

Nursing is a means to an end. Once I have my permanent residency, I’ll go back to university, maybe study business or law—something that commands respect back home and will make my family proud. For now, I’ll keep my nursing ambitions quiet. It’s a temporary sacrifice for a long-term gain.—Ruvin

Ruvin’s perspective further emphasizes the calculated nature of these choices, highlighting the willingness of these students to make temporary sacrifices in pursuit of long-term goals. He is consciously navigating different cultural contexts, adapting his aspirations to meet the expectations of his home country while leveraging the opportunities available in Australia. This exemplifies these students’ complex identity work as they negotiate their roles and aspirations across borders.

## “Nurse,” a Word Unspoken: Linguistic Camouflage and the Quest for Cultural Acceptance

The term “nurse” carries significant cultural baggage for male South Asian students, prompting them to employ linguistic camouflage even when their families are aware of their chosen profession. This avoidance of the word “nurse” is a further example of the identity work these students engage in as they navigate the complexities of their professional identity in relation to cultural norms and expectations. This also reflects the concept of linguistic framing, where the choice of words is strategically employed to shape perceptions.

It’s not about deceiving my family; they know I’m studying to be a nurse. But the word itself carries a stigma, a sense of emasculation back home. It’s just easier to be broad and say “health science.”—Raj

Raj’s statement highlights the stigma associated with the word “nurse” in his cultural context, suggesting that it is perceived as incompatible with traditional notions of masculinity. His choice to use the broader term “health science” is a conscious effort to mitigate this stigma and maintain a sense of masculine identity within his family.

I’m proud of my choice to become a nurse, but I’m also aware of the cultural barriers I face because of my choice. It is common for other friends studying nursing to do the same. They do not utter the word “nurse” in front of extended friends and families when a conversation ensues regarding study and career trajectory in Australia.—Amir

Amir’s observation reveals that this linguistic strategy is not an isolated practice but a shared coping mechanism among male South Asian nursing students. This collective avoidance of the word “nurse” underscores the pervasive nature of the cultural stigma and the need for these students to negotiate their professional identity within their social circles constantly. This shared strategy can be seen as a form of collective identity work, where a group of individuals facing similar challenges develop shared strategies to navigate their marginalized identity.

I’m not just studying “nursing”; I’m becoming a healer, a caregiver, a vital part of the healthcare team. It’s time we redefine what it means to be a man in the 21st century. Nursing is a strength, not a weakness. It’s compassion, not subservience.—Ishaan

Ishaan’s powerful statement challenges the traditional gender roles and stereotypes that underpin the stigma surrounding nursing. He actively reframes the profession, emphasizing its positive attributes and advocating for a redefinition of masculinity that embraces caregiving and compassion. This represents a form of resistance against dominant cultural norms and an attempt to reconstruct his professional identity on his own terms.

## Discussion

The present study reveals a complex narrative, showcasing the intricate interplay of cultural expectations, migration aspirations, and the pursuit of personal identity among male international students from the South Asian diaspora studying nursing. These students navigate a delicate tightrope between adhering to traditional gender roles and forging their own paths in a profession that offers both challenges and opportunities. Their experiences underscore the deeply ingrained societal perceptions of nursing as a “feminine” domain, compelling them to adopt linguistic camouflage and strategic career planning to reconcile their aspirations with cultural norms. Furthermore, the study uncovers the multifaceted motivations driving their career choices, extending beyond migration opportunities and economic considerations to encompass personal freedom, the fulfillment of broader ambitions, and the quest for self-acceptance.

The strategic use of nursing as a pathway to migration and economic stability, a phenomenon well-documented in existing literature (Konlan et al., 2023; Smith et al., 2022; Villamin et al., 2023), takes on a new dimension when viewed through the lens of cultural dissonance and gendered expectations. While global nursing shortages and the demand for skilled health care professionals in developed countries undoubtedly drive this trend, this study reveals a compelling narrative of individual agency and calculated risk-taking among male South Asian international students. Their decision to pursue nursing, a profession often incongruent with their cultural norms and masculine ideals (Cho & Jang, 2021), highlights a fascinating paradox: they leverage a traditionally female-dominated field to achieve social mobility and economic security in a new country. This strategic maneuvering can be seen as a form of “cultural arbitrage,” wherein individuals capitalize on the discrepancies between their cultural capital and the opportunities available in a different context (Hu, 2023). This concept, borrowed from the field of economics, offers a fresh perspective on the motivations of these students, who effectively “trade” their cultural background and willingness to challenge norms for the potential rewards of migration and professional advancement.

The internal conflict experienced by these students as they balance personal aspirations with familial and societal expectations speaks to the enduring power of cultural norms and the complexities of challenging traditional gender roles. Their narratives highlight the emotional labor involved in navigating these complexities and the resilience they demonstrate in pursuing their chosen paths. This negotiation can be effectively understood through the lens of identity work, where individuals actively shape and reshape their identities in response to social pressures and personal desires (Caza et al., 2018). For instance, participants like Arun and Farhan actively redefined their professional identities by using the term “health science,” while Ruvin and Rohan viewed nursing as a stepping stone to more culturally accepted professions. Specifically, these students engage in a delicate balancing act, striving to maintain their sense of masculinity while embracing a profession often associated with femininity. This balancing act is further complicated by the transnational context, as these students are negotiating their identities across different cultural landscapes (Belford & Lahiri-Roy, 2019; Binah-Pollak & Yuan, 2022; Kasun et al., 2022). Moreover, the pursuit of personal freedom and self-acceptance, as evidenced by some participants’ desire to escape the constraints of their home countries, adds another layer of complexity to their identity work. This aligns with research highlighting the experiences of marginalized groups who use migration as a strategy for self-discovery and liberation (Usta & Ozbilgin, 2023). This study advances the discourse by revealing the diverse experiences within this group, including those who strategically choose nursing precisely because of its perceived inclusivity and acceptance of gender diversity, as seen in Kamal’s narrative but also reflected in the motivations of other participants who sought a more tolerant environment. This nuanced understanding challenges the simplistic notion of a singular “male nursing student experience” and highlights the intersectionality of gender, sexuality, and cultural identity. It also speaks to the potential of nursing to become a site of resistance and transformation, where individuals can challenge traditional norms and create spaces for greater inclusivity and self-expression.

Moreover, the findings raise critical questions about the limitations of linguistic camouflage and the urgent need for a broader societal shift in perceptions of nursing. While strategic reframing may offer a temporary shield against cultural judgment, it can also inadvertently perpetuate the very stereotypes it seeks to avoid. Challenging hegemonic masculinity, as Liu et al. (2022) argue, requires not only individual resistance but also a transformation of social structures and cultural norms. By resorting to linguistic camouflage, male South Asian nursing students may unintentionally reinforce the notion that nursing is inherently feminine and incompatible with masculine ideals. This aligns with research on the power of language to shape perceptions and reinforce stereotypes (Formanowicz & Hansen, 2022). Concealing their true profession can be seen as a form of “symbolic violence” (Schubert, 2012), where individuals internalize and reproduce the dominant cultural norms that marginalize them. Furthermore, existing recruitment strategies in health care, which often rely on mobilizing specific versions of masculinity to attract men to the profession (Cottingham, 2014), may inadvertently contribute to the problem. Instead of fundamentally challenging hegemonic masculinity, these approaches may simply co-opt it, presenting a narrow and potentially exclusionary vision of what it means to be a male nurse (Cottingham, 2019). Therefore, dismantling the deeply ingrained gendered stereotypes surrounding nursing requires a multi-pronged approach that encompasses both individual and collective action alongside institutional efforts to promote gender equality (Teresa-Morales et al., 2022). This necessitates a shift in cultural attitudes toward caregiving professions, particularly within South Asian communities, recognizing them as valuable and suitable for all genders. This shift also extends globally, as many high-income liberal democracies grapple with similar gendered expectations in health care, highlighting the need for a broader re-evaluation of caregiving professions across diverse cultural contexts (Milner et al., 2021). Achieving this transformation calls for a critical examination of the language used to describe nursing, the images used to represent it, and a conscious effort to dismantle the power dynamics that perpetuate its gendered construction. Creating truly inclusive spaces within nursing requires challenging individual biases and the broader societal norms and structures that reinforce them.

This study reveals a compelling paradox within the power dynamics of nursing education, particularly for male students from non-Western backgrounds. While nursing globally is a primarily female-dominated field, these men often experience a unique form of marginalization (Huang, 2024). They are simultaneously hypervisible, as their presence as men in a female-dominated field attracts attention and scrutiny, yet often remain invisible, with their unique perspectives and challenges being overlooked or dismissed (Raghavan et al., 2023; Yokoya et al., 2023). This experience of hypervisibility and invisibility creates complex challenges, particularly for students from South Asian backgrounds. Their experiences are shaped not only by gender but also by the intersection of their cultural identities and the dynamics of being an international student in a new cultural environment. For instance, their accents, cultural practices, or even their names can become markers of difference, leading to increased scrutiny or stereotyping. This aligns with research on the experiences of minority groups in predominantly white spaces, where individuals often report feeling both hypervisible and invisible, their presence simultaneously highlighted and ignored (Jackson et al., 2022; Subu et al., 2022). This paradoxical experience can be conceptualized as a form of “double consciousness” (Joseph & Golash-Boza, 2021), where individuals are forced to navigate their identity through the lens of both their own cultural background and the dominant societal norms. This concept, initially developed by Du Bois (1903) to describe the experiences of African Americans, provides a valuable framework for understanding how individuals from marginalized groups experience a sense of duality and are constantly aware of how the dominant culture perceives them. While previous research has explored the paradoxical position of male nurses in general (Evans, 1997; Kellett et al., 2014; Salvador & Alanazi, 2024), this study extends these findings by highlighting the specific experiences of South Asian male nursing students and linking their experiences to the concept of double consciousness. These students not only navigate the general challenges faced by men in a female-dominated profession but also the additional complexities of being international students from a distinct cultural background.

Furthermore, the study highlights the hostile environment that male students often perceive in nursing education. Some participants reported experiencing a hostile environment in their clinical placements, marked by subtle and overt forms of discrimination. For instance, Raj described an incident where a preceptor repeatedly criticized his accent, making him feel marginalized and excluded. This experience highlights the additional barriers faced by international students, who may be judged based on their communication style rather than their clinical competence. This finding is consistent with research that highlights the challenges faced by international students in health care settings, including language barriers, cultural misunderstandings, and discrimination (Oduwaye et al., 2023; Pandey et al., 2021). Furthermore, studies have documented gender bias and unsupportive behaviors from educators in nursing education, which create significant obstacles for male students (Hosseini et al., 2022). This interplay of factors underscores the need for a more nuanced and intersectional approach to understanding the experiences of male South Asian nursing students and other students from diverse linguistic and cultural backgrounds. Creating educational environments that are truly inclusive and supportive of all learners requires a commitment to addressing not only overt prejudice but also the subtle, often unintentional biases that can create barriers to success.

In conclusion, this study has illuminated the intricate pathways navigated by male South Asian international nursing students in Australia, revealing the dynamic interplay of cultural expectations, personal aspirations, and strategic decision-making that characterizes their journeys. The findings underscore the significant influence of cultural perceptions of nursing, compelling these students to employ linguistic camouflage and navigate gendered stereotypes while also highlighting the resilience and adaptability they demonstrate in pursuing their professional goals. The authors acknowledge that their positionality as former international students and nurses from South and East Asian backgrounds may have influenced their interpretation of the data. However, they maintain that this insider perspective also provided valuable insights into the nuances of the participants’ experiences. Ultimately, this research highlights the critical need for nursing education and practice to move beyond simplistic notions of cultural difference and to engage with the complex realities of individuals navigating multiple cultural identities and systemic barriers within the profession.

## Implications for Transcultural Nursing

This study’s findings have significant implications for transcultural nursing research, education, and policy. The experiences of male South Asian international nursing students, particularly their use of linguistic camouflage and navigation of cultural expectations, highlight the need for further investigation into the intersection of gender, culture, and professional identity within nursing. Future research should explore the long-term career trajectories of these students and the effectiveness of culturally tailored interventions. Longitudinal studies are crucial to understanding how these students’ experiences in nursing school shape their professional development and career choices, informing a more culturally sensitive approach to nursing recruitment and retention. Nursing education programs must actively foster inclusivity and challenge traditional gender stereotypes. This requires curriculum reform to incorporate diverse perspectives on gender and caregiving, showcasing examples of male nurses from various cultural backgrounds in diverse roles. Tailored support programs, including mentorship opportunities with individuals who share similar cultural experiences, are essential for helping these students navigate cultural challenges, build a sense of belonging, and develop effective coping mechanisms. Faculty training in cultural competence is crucial, with a focus on developing awareness of unconscious biases, promoting inclusive teaching practices, and creating a safe learning environment. Furthermore, policies should address systemic barriers faced by international students, promoting greater inclusion in clinical placements and advocating for equitable opportunities. This could involve initiatives to educate clinical preceptors on implicit bias and culturally sensitive communication, as well as establishing clear reporting mechanisms for students who experience discrimination. By implementing these changes, nursing education can empower all students, regardless of gender or cultural background, to thrive and contribute to a more culturally competent and responsive health care workforce.
